# Bad Neighbors: Arsenic-Induced Tumor Cells Convert Normal Stem Cells into a Cancerous Phenotype

**DOI:** 10.1289/ehp.120-a244a

**Published:** 2012-06-01

**Authors:** Julia R. Barrett

**Affiliations:** Julia R. Barrett, MS, ELS, a Madison, WI–based science writer and editor, has written for *EHP* since 1996. She is a member of the National Association of Science Writers and the Board of Editors in the Life Sciences.

Millions of people are exposed to inorganic arsenic, a human carcinogen, in drinking water. *In vitro* and animal studies have shown that malignant cell lines and tumors induced by exposure to inorganic arsenic include more cancer stem cells than other tumor types. Arsenic is known to transform normal stem cells into cancer stem cells, but a new study shows that arsenic-induced malignant cells may do so even without direct arsenic exposure [*EHP* 120(6):865–871; Xu et al.]. This may explain why arsenic-induced tumors contain so many cancer stem cells.

In normal tissues, stem cells are the source of replacements for damaged or dead cells. Similarly, cancer stem cells are a source of new malignant cells that allow tumors to grow and spread, and they are integral to tumor initiation, progression, and metastasis. The cells’ origins and formation are not entirely clear, but evidence suggests they may transform from normal stem cells under the influence of inflammatory cytokines and other factors.

With the prostate identified as a possible target tissue of arsenic, the authors of the current study created malignant epithelial cells (MECs) by exposing a normal human prostatic epithelial cell line, RWPE-1, to sodium arsenite. The RWPE-1 line was also used as the source for normal stem cells, which were co-cultured with either control RWPE-1 cells or MECs. A semipermeable membrane between the cell types in the co-culture system prevented direct cell-to-cell contact but allowed secreted factors to pass freely between cells. The system contained no detectable arsenic.

During the weeks of co-culture and afterward, the investigators assessed the development of a cancer stem cell phenotype, which was defined by the secretion of matrix metalloproteinases (MMPs). Cellular gene expression was assessed at both the transcription and translation levels to determine which factors were involved in the changed phenotype. Finally, normal stem cells cultured alone were treated with interleukin-6 (an inflammatory cytokine secreted by MECs) and then analyzed for MMP activity and gene expression.

Test results indicated that normal stem cells in noncontact co-culture with MECs developed a cancer stem cell phenotype. MMP activity and invasive capacity increased, while *PTEN* tumor suppressor gene expression decreased, similar to previous observations in prostatic cancer stem cells. Changing patterns of gene expression over time, including early loss and later reactivation of several genes, were consistent with changes observed in cancer stem cells during oncogenesis. Similar MMP and gene-expression changes were induced in normal stem cells cultured alone and treated with interleukin-6, suggesting that inflammatory cytokines produced by malignant cells might cause the transformation to cancer stem cells.

The results suggest that MECs may recruit normal stem cells to become cancer stem cells, potentially affecting the growth and spread of cancer, and that interleukin-6 may be one of the recruitment signals. It is unknown whether MECs transformed by carcinogens other than arsenic have a similar ability to alter normal stem cells; more investigation of this mechanism is needed.

